# ASC-Exosomes Ameliorate the Disease Progression in SOD1(G93A) Murine Model Underlining Their Potential Therapeutic Use in Human ALS

**DOI:** 10.3390/ijms21103651

**Published:** 2020-05-21

**Authors:** Roberta Bonafede, Ermanna Turano, Ilaria Scambi, Alice Busato, Pietro Bontempi, Federica Virla, Lorenzo Schiaffino, Pasquina Marzola, Bruno Bonetti, Raffaella Mariotti

**Affiliations:** 1 Department of Neuroscience, Biomedicine and Movement Sciences, University of Verona, 37134 Verona, Italy; roberta.bonafede@univr.it (R.B.); ermanna.turano@univr.it (E.T.); ilaria.scambi@univr.it (I.S.); federica.virla@univr.it (F.V.); lorenzo.schiaffino@univr.it (L.S.); 2 Department of Computer Sciences, University of Verona, 37134 Verona, Italy; alice.busato@univr.it (A.B.); pietro.bontempi@univr.it (P.B.); pasquina.marzola@univr.it (P.M.); 3 Neurology Unit, Azienda Ospedaliera Universitaria Integrata Verona, 37126 Verona, Italy; bruno.bonetti@univr.it

**Keywords:** amyotrophic lateral sclerosis, stem cells, extracellular vesicles, motoneurons, neuromuscular junction, homing, MRI

## Abstract

Amyotrophic lateral sclerosis (ALS) is a fatal neurodegenerative disease characterized by progressive degeneration of motoneurons. To date, there is no effective treatment available. Exosomes are extracellular vesicles that play important roles in intercellular communication, recapitulating the effect of origin cells. In this study, we tested the potential neuroprotective effect of exosomes isolated from adipose-derived stem cells (ASC-exosomes) on the in vivo model most widely used to study ALS, the human SOD1 gene with a G93A mutation (SOD1(G93A)) mouse. Moreover, we compared the effect of two different routes of exosomes administration, intravenous and intranasal. The effect of exosomes administration on disease progression was monitored by motor tests and analysis of lumbar motoneurons and glial cells, neuromuscular junction, and muscle. Our results demonstrated that repeated administration of ASC-exosomes improved the motor performance; protected lumbar motoneurons, the neuromuscular junction, and muscle; and decreased the glial cells activation in treated SOD1(G93A) mice. Moreover, exosomes have the ability to home to lesioned ALS regions of the animal brain. These data contribute by providing additional knowledge for the promising use of ASC-exosomes as a therapy in human ALS.

## 1. Introduction

Amyotrophic lateral sclerosis (ALS) is a fatal neurodegenerative disease characterized by the progressive degeneration of motoneurons (MN) in the primary motor cortex, brainstem, and spinal cord [1]. The disease is sporadic in 90%–95% of cases, while it is familial in the remaining cases (with an autosomal dominant, autosomal recessive, or X-linked inheritance), and 20% of the familial ALS are due to mutations in the gene encoding for Cu^2+^/Zn^2+^ superoxide dismutase (SOD1) [2]. The mechanisms underlying the neurodegeneration of the disease are complex and still need to be clarified [3].

Transgenic mice overexpressing the human SOD1 gene with a reported G93A mutation (SOD1(G93A)) is the most widely used murine model to study ALS. This model exhibits the clinical signs and the pathological features of human patients, and provides an optimal in vivo model for investigating the pathogenesis of disease and for testing new therapeutic approaches [4].

Due to the complexity of the pathogenetic mechanisms involved in this neurodegenerative disorder, to date, there is no effective treatment available [5]. A promising therapeutic approach in neurodegenerative diseases is represented by stem cells that are able to self-renew, differentiate, and home to damage sites, which contribute to tissue repair and regeneration. Mesenchymal stem cells (MSC) are considered to be the best candidates and, among these, adipose-derived stem cells (ASCs) are accessible in large amounts and allow an easily autologous cell transplantation [6].

Several studies, including ours, have already demonstrated the beneficial effects of stem cells after their transplantation in murine models of ALS. The cells delayed the symptom progression of the disease, improved the function of the neuromuscular junction, and decreased the inflammatory response of the animals, indicating that they could promote neuroprotection [7,8,9,10]. However, despite the encouraging results concerning stem cell treatment, migration, engraftment, and differentiation of stem cells at the sites of injury were reported rarely. For these reasons, the hypothesis that stem cells exert their therapeutic activity through secreted molecules and extracellular vesicles is plausible [3,11]. Among extracellular vesicles, exosomes are 50–150 nm in diameter and play important roles in intercellular communication, recapitulating the effect of stem cell transplantation by transferring biologically active molecules to recipient cells, altering their gene expression and behavior [12]. Several studies have suggested that exosomes isolated from stem cells are involved in neuronal protection, nerve regeneration, neurological recovery, and synaptic plasticity. These vesicles can be exploited as a therapy instead of their derived parental cells, avoiding the limitations and risks associated with cell transplantation (reviewed in [13]).

In the present study, we tested the neuroprotective effect of exosomes isolated from ASCs (ASC-exosomes) in the SOD1(G93A) murine model of ALS. Moreover, we compared the effect of two different routes of exosomes administration, i.e., intravenous (i.v.) and intranasal (i.n.). First, we administered ASC-exosomes i.v., as previously done with ASCs [8]. Furthermore, we assessed the i.n. delivery, since it is easily transferable to ALS patients avoiding their hospitalization and represents a noninvasive way to deliver exosomes directly to the CNS.

In this study, we demonstrated, for the first time, that repeated administration of exosomes (i.v. or i.n.) improved the motor performance; protected the lumbar MN, neuromuscular junction (NMJ), and muscle; and decreased the glial cells activation in treated SOD1(G93A) mice. Moreover, we tested the capacity of exosomes to reach the CNS and the lesion sites. For this purpose, we used magnetic resonance imaging (MRI) and exosomes labeled with ultra-small superparamagnetic iron oxide nanoparticles (USPIO) as contrast agents to produce a detectable change in signal intensity [14]. After i.n. administration, we detected labeled exosomes (exosomes-USPIO) in the brain, indicating that the vesicles reached the CNS and accumulated in the typical lesioned brainstem motor nuclei of the SOD1(G93A) mice.

Taken together, these results prove that ASC-exosomes are able to slow down the clinical progression of ALS; exert a protective effect on MN, NMJ, and muscle; and reduce glial cells activation. Moreover, ASC-exosomes home to lesioned regions of animal brain. These data contribute by providing additional knowledge for the promising use of ASC-exosomes as a novel cell-free therapy in ALS.

## 2. Results

### 2.1. Isolation and Characterization of ASC-Exosomes

**Figure 1:**
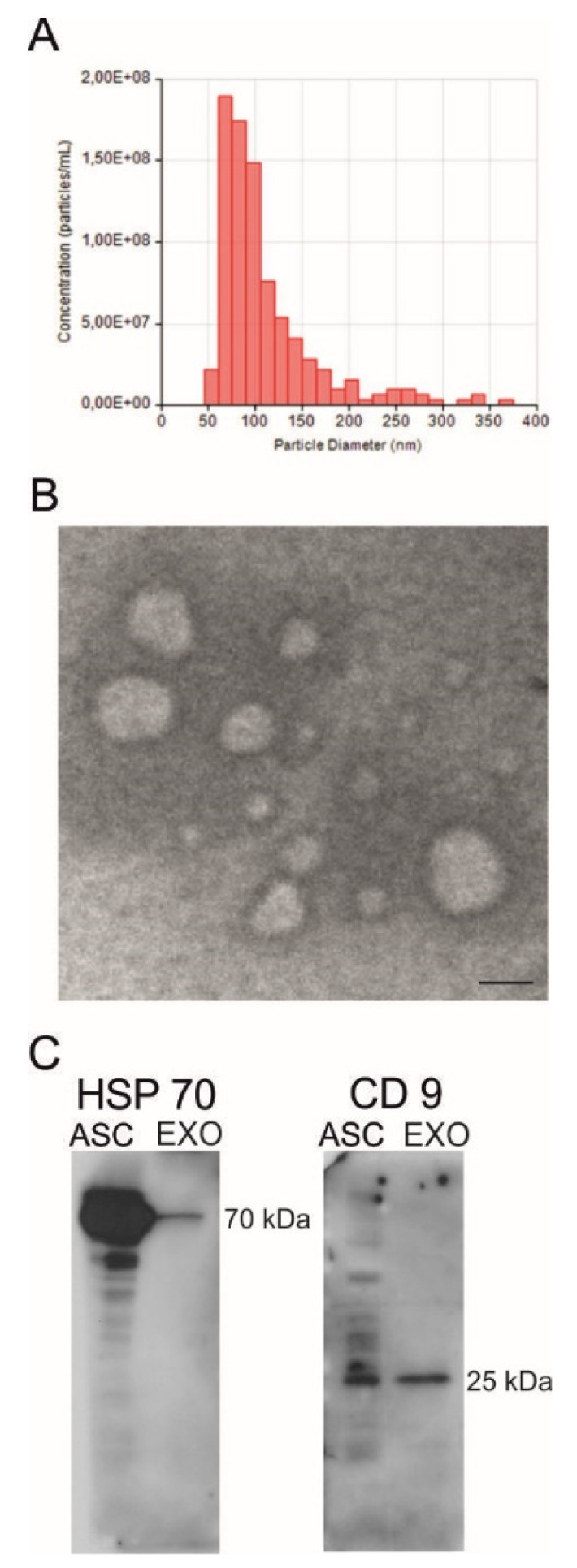
Characterization of exosomes isolated from adipose-derived stem cells (ASC-exosomes). (**A**) Histogram of the concentration and the particle diameter of ASC-exosomes; (**B**) Electron microscopy shows vesicles with characteristic morphology and size of exosomes. Scale bar, 100 nm; (**C**) The blots show Western blot detection of the expression of HSP70 (70 kDa) and CD9 (25 kDa) in ASC-exosomes (EXO). Adipose-derived stem cell (ASC) lysates (ASC) were used as a positive control.

Exosomes were isolated from ASC supernatant with an exosome isolation kit. The protein concentration was quantified and the yield of proteins for each isolation was about 200 µg/mL. The concentration of exosomes was 6–8 × 10^8^ particles/mL, with a particle diameter mean of 110 nm (Figure 1A). Ultrastructure analysis of the exosomes by TEM revealed round vesicles with lipid bilayers with a diameter of 50 to 150 nm (Figure 1B). Western blot analysis revealed specific exosomal markers, such as HSP70 (70 kDa) and CD9 (25 kDa) (Figure 1C). These results confirm that size, morphology, and the presence of specific protein markers are consistent with exosome extracellular vesicles.

### 2.2. ASC-Exosomes Administration Improves Motor Performance of SOD1(G93A) Mice

In order to test the possible beneficial effect of ASC-exosomes in SOD1(G93A) mice, specific motor tests were performed to evaluate the disease progression in exosomes- and PBS-treated mice. Furthermore, we tested two different routes of exosomes administration, i.v. and i.n.

**Figure 2:**
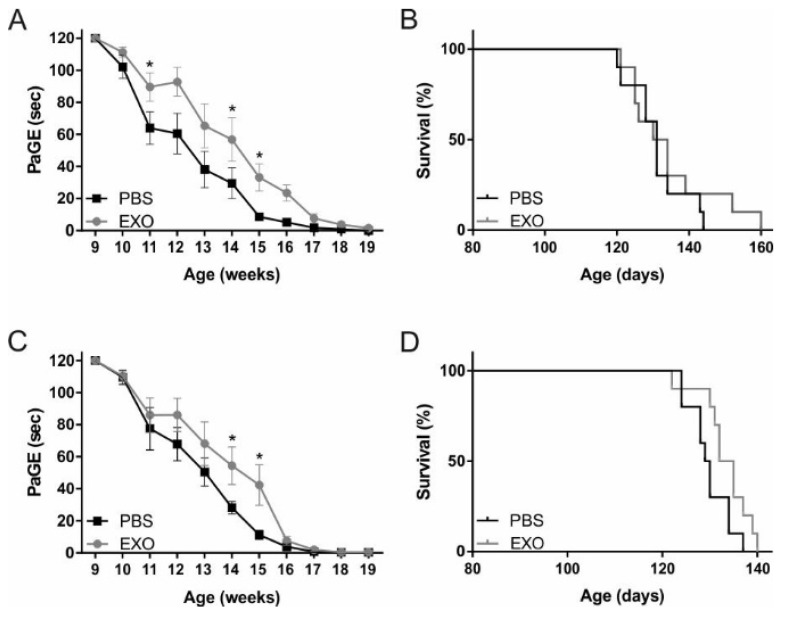
Motor performances and survival of human SOD1 gene with a G93A mutation (SOD1(G93A)) mice treated intravenously (**A**, **B**) and intranasally (**C**, **D**). (**A**, **C**) The graphs show the motor performances of SOD1(G93A) mice treated with PBS (black line) or with ASC-exosomes (EXO, grey line). The paw grip endurance (PaGE) test shows a global improvement of motor performance of the EXO-treated mice as compared with the PBS group, with significant differences at 11, 14, and 15 weeks (* *p* = 0.0494, * *p* = 0.0308 and * *p* = 0.0102, respectively) with i.v. treatment (**A**) and significant differences at 14 and 15 weeks (* *p* = 0.0219 and * *p* = 0.0431, respectively) with i.n. treatment (**C**). Data are reported as mean ± SEM. (**B**, **D**) The graphs show the survival rate of SOD1(G93A) mice treated with PBS (black line) or with ASC-exosomes (EXO, grey line). Graphs show the percentages of occurrence of the events.

We administered ASC-exosomes intravenously in order to compare their effect with that obtained with ASCs [8] and demonstrated that ASCs act through the release of extracellular vesicles. The i.v. administration of ASC-exosomes was started from the clinical onset of pathology (defined in the Material and Method section) and determined an improvement of the grip strength of the mice, monitored with the paw grip endurance (PaGE) test (Figure 2A). The graph shows that the mice started to fail the test at the beginning of 10 weeks of life, which was the time when they started to receive ASC-exosomes injections. The performance of the mice progressively declined during the following weeks, when the mice treated with exosomes showed a better performance as compared with the PBS group, with a statistically significant difference at 11, 14, and 15 weeks of life (*p* = 0.0494, *p* = 0.0308, and *p* = 0.0102, respectively) (Figure 2A). The improvement of motor performance after administration of ASC-exosomes was comparable with ASC treatment [8].

In order to assess the effect of an alternative route of administration of ASC-exosomes, the mice were treated intranasally from the beginning of 10 weeks of life (clinical onset of disease) until the end stage (19 weeks of life). An improvement of the grip strength of the exosomes-treated mice was obtained as compared with the PBS group, with a statistically significant difference at 14 and 15 weeks of life (*p* = 0.0219 and *p* = 0.0431, respectively) (Figure 2C).

The PaGE test showed that the beneficial effect of ASC-exosomes persists for six weeks and the motor performance progression was similar, irrespective of the route of delivery. In both cases, the beneficial effect observed in ASC-exosomes treated mice disappeared around week 17 of life, whereas no differences were reported between exosomes- and PBS-treated mice (Figure 2A,C).

Concerning the rotarod test, no significant difference was observed among the mice that received ASC-exosomes or PBS with different routes of administration (i.v and i.n.) It is possible that we did not observe any differences since the motor coordination results evaluated by the rotarod test usually altered in the late phase of the disease, at the time when the ASC-exosomes reduced their efficacy in terms of muscle strength (as shown by the PaGE test).

The graphs regarding the survival of the mice show that both treatments exerted no significant effect, although some exosomes-treated mice had a prolonged lifespan as compared with the PBS-treated mice (Figure 2B,D).

### 2.3. ASC-Exosomes Administration Protects Lumbar Spinal Cord MN from Neurodegeneration

**Figure 3:**
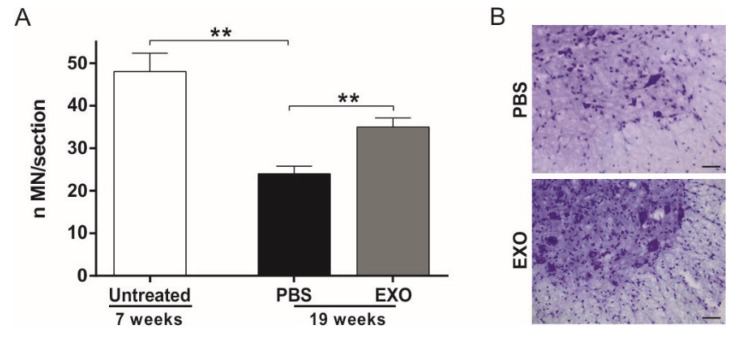
Effect of i.v. ASC-exosomes treatment on motoneurons (MN) survival in SOD1(G93A) mice. (**A**) The graph shows the significant progressive loss of MN during the disease progression (as compared with untreated mice at week 7 and PBS-treated mice at the end stage of the disease, ** *p* = 0.0026). The i.v. treatment with ASC-exosomes (EXO) significantly increases the MN survival in the lumbar section (L1–L5) of the spinal cord as compared with the PBS-treated group (** *p* = 0.0078). Data are shown as mean ± SEM; (**B**) Representative Nissl staining of the lumbar spinal cord MN of PBS- and ASC-exosomes (EXO) treated mice. Note that a higher MN number in the mice that receive exosomes treatment as compared with the PBS group. Magnification 20×, scale bar 50 µm.

**Figure 4:**
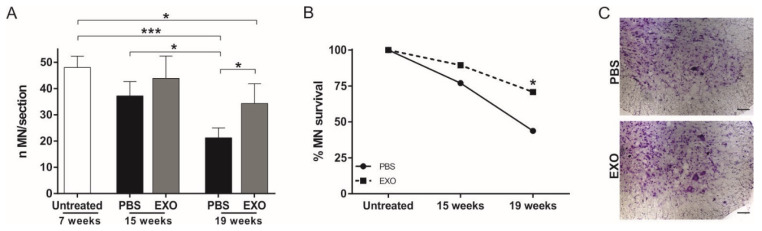
Effect of i.n. ASC-exosomes treatment on MN survival in SOD1(G93A) mice. (**A**) The graph shows the significant progressive loss of MN during the disease progression. At the end stage of the disease (19 weeks of life), the i.n. treatment with ASC-exosomes (EXO) significantly increases the MN survival in the lumbar section (L1–L5) of the spinal cord as compared with the PBS-treated group (* *p* = 0.034). Data are reported as mean ± SEM (untreated versus PBS 19 weeks *** *p* = 0.001; untreated versus EXO 19 weeks * *p* = 0.049; PBS 15 weeks versus PBS 19 weeks * *p* = 0.049); (**B**) The graph shows the percentage of MN during the disease progression. Note, the ability of ASC-exosomes to protect MN from death. Data are reported as the percentage of surviving MN (* *p* = 0.034); (**C**) Representative Nissl staining of the lumbar spinal cord MN of the PBS and ASC-exosomes (EXO) treated mice. Note, a higher MN number in mice that receive exosomes treatment as compared with the PBS group. Magnification 20×, scale bar 50 µm.

To evaluate the neuroprotective effect of ASC-exosomes in the SOD1(G93A) mice, the stereological count of lumbar MN was performed on sections from L1–L5 metamers of the spinal cord. To evaluate the MN loss during the disease progression, a third group of untreated SOD1(G93A) mice was sacrificed at the preclinical stage of the disease (week seven). A significant loss of MN was observed in PBS-treated mice at 19 weeks of life, with both routes of treatment (with *p* = 0.0026 in the i.v. treatment and *p* = 0.001 in the i.n. treatment) showing a progressive loss of MN of about 50% due to the disease progression as compared with untreated mice sacrificed at seven weeks of life (Figure 3A and Figure 4A). The i.v. ASC-exosomes administration determines a significant increase in surviving MN as compared with the PBS mice at the end-stage of the disease (19 weeks, *p* = 0.0078) (Figure 3A). This data indicates that ASC-exosomes are able to protect MN from death, as shown in a representative image of PBS- and exosomes-treated mice (Figure 3B).

The same neuroprotective effect was observed after i.n. administration of ASC-exosomes at 19 weeks of life (*p* = 0.034, Figure 4A).

For the i.n. administration, an additional group of mice was used. This group received ASC-exosomes from the onset of the clinical sign until week 15 of life, which was the time when the mice were sacrificed. Week 15 represents the time point when the difference in the motor performance of the mice was statistically significant for the PBS-treated versus exosomes-treated groups (see Figure 2C).

At week 15, we did not observe a significant difference in the number of lumbar MN in the exosomes-treated as compared with the PBS-treated mice. Moreover, the MN number of untreated mice, at the preclinical stage of the disease (week seven) as compared with PBS-treated mice, was not significantly decreased (Figure 4A). However, the PBS-treated mice sacrificed at week 15 of life showed 22.9% MN loss as compared with the untreated mice at the preclinical stage of the disease (week seven), whereas the exosomes-treated mice showed a lower percentage of MN loss (10.4%) than the PBS-treated mice, which underlines the effect of exosomes also at this stage of disease (Figure 4B). Finally, the progressive MN death during the disease course in the PBS-treated mice reached 56.2% after 19 weeks of life, whereas, in the exosomes-treated mice, the percentage of MN death was 26.7% (*p* = 0.034, Figure 4B,C).

Altogether, these results indicate that ASC-exosomes treatment is able to protect the lumbar spinal cord MN from degeneration.

### 2.4. ASC-Exosomes Administration Preserves Neuromuscular Junction Functionality and Skeletal Muscle Fiber Morphology

**Figure 5:**
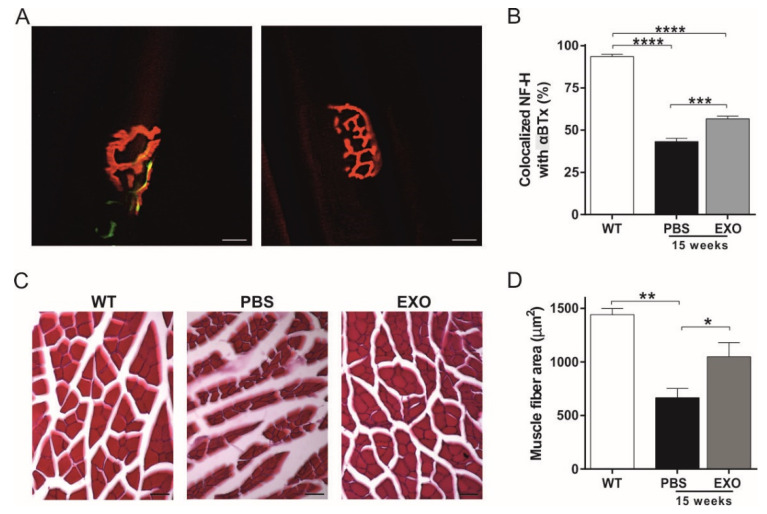
Effect of i.n. administration of ASC-exosomes on skeletal muscle of SOD1(G93A) mice. (**A**) Representative images of the correct architecture of the neuromuscular junction (NMJ), in which a colocalization of presynaptic NF-H (green) and αBTx (red) is showed (left). On the right is reported the degeneration of the NMJ, in which non colocalization between NF-H (green) and αBTx (red) is reported, due to presynaptic loss of neurofilament that leave to denervation. Magnification 40×, scale bar 25 µm; (**B**) The graph shows a significant decrease in colocalization of NF-H and αBTx in SOD1(G93A) mice at 15 weeks of life as compared with the wild-type (WT) mice (**** *p* < 0.0001 comparing WT mice with both PBS- or exosomes-treated SOD1(G93A) mice). Note that ASC-exosomes treated mice (EXO) showed a significant increase in NMJ with presynaptic terminals colocalized with αBTx as compared with the PBS group (*** *p* = 0.0006). Data are shown as mean ± SEM; (**C**) Representative images of hematoxylin-eosin (HE) staining in the WT mice and the SOD1(G93A) mice treated with PBS or ASC-exosomes (EXO) at 15 weeks of life. Note, the reduced degeneration of the skeletal muscle in ASC-exosomes treated mice. Magnification 20×, scale bar 50 µm; (**D**) The graph shows the quantitative analysis of the average fiber area in the groups of mice. A significant decrease in fiber area in the PBS-treated SOD1(G93A) mice at 15 weeks of life was detected as compared with the WT mice (** *p* = 0.0034). Note that ASC-exosomes treated mice (EXO) showed a significant increase in fiber area as compared with the PBS group (* *p* = 0.0410). Data are shown as mean ± SEM.

As reported above, the mice treated with ASC-exosomes showed a significant improvement in motor performance as compared with the PBS-treated mice at 14 and 15 weeks of life. Since this data were not supported by a higher significant MN survival of exosomes-treated animals at these intermediate time points, we analyzed the NMJ and the skeletal muscle fibers in exosomes- and PBS-treated mice at 15 weeks of life. The NMJ with complete or partial colocalization of presynaptic NF-H and the postsynaptic α-bungarotoxin (αBTx) were defined as innervated, while the total loss of colocalization indicated the degeneration of the NMJ (Figure 5A). At 15 weeks of life, a significant difference (*p* < 0.0001) of innervated NMJ was reported in wild-type (WT) mice as compared with the SOD1(G93A) PBS- or exosomes-treated mice, indicating the hindlimb NMJ degeneration of the SOD1(G93A) mouse model. Interestingly, the SOD1(G93A) mice treated with ASC-exosomes showed a higher innervated NMJ number as compared with PBS-treated mice (*p* = 0.0006) (Figure 5B). This result indicates that ASC-exosomes treatment preserves the NMJ, slowing down the degeneration of MN and, consequently, the detachment of the axons from the muscle.

In addition to the NMJ, we analyzed the morphological changes of the gastrocnemius muscle (Figure 5C). Compared to the WT mice, hematoxylin-eosin (HE) staining of PBS-treated mice showed enhanced endomysial space, degenerated fibers with reduced diameter, and a significantly decreased muscular fiber area (Figure 5C,D, *p* = 0.0034). Interestingly, the ASC-exosomes treatment attenuated the degeneration of fibers with an average muscular fiber area significantly increased as compared with the PBS group (*p* = 0.0410), and without significant differences with the WT mice (Figure 5C,D).

### 2.5. Effect of ASC-Exosomes Administration on Glial Cells

**Figure 6:**
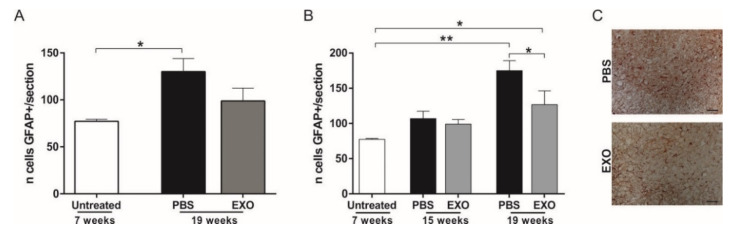
Effect of i.v. and i.n. ASC-exosomes treatment on astrocytosis in SOD1(G93A) mice. (**A**) The graph shows the number of GFAP^+^ cells in the lumbar section (L1–L5) of the spinal cord, comparing untreated with i.v. treated SOD1(G93A) mice. Note, the progressive increase of astrocytosis during the disease progression, and a trend in ASC-exosomes (EXO) treatment to decrease astrocyte activation (* *p* = 0.05); (**B**) The graph shows the number of GFAP^+^ cells in the lumbar section (L1–L5) of the spinal cord, comparing untreated with i.n. treated SOD1(G93A) mice sacrificed at 15 and 19 weeks of life. At the end stage of the disease, note, a significant difference in decreasing astrocyte activation in ASC-exosomes treated mice (EXO) as compared with the PBS group (* *p* = 0.03 ** *p* = 0.006); (**C**) Representative staining of the lumbar spinal cord of the PBS and ASC-exosomes (EXO) treated mice. Note, a lower number of GFAP^+^ cells in the ASC-exosomes treated mice as compared with the PBS-treated mice. Magnification 20×, scale bar 50 µm.

Analysis of glial cells was performed on the lumbar section of the spinal cord of exosomes- and PBS-treated mice. We found a progressive increase in astrocyte activation during the disease course (comparing mice at the preclinical stage of the disease to the PBS-treated mice and at 15 and 19 weeks), which reached a significant difference at 19 weeks for both i.v. and i.n. treatments (*p* = 0.05 and *p* = 0.006, respectively, Figure 6A,B).

In mice treated intravenously, a tendency to downregulate the GFAP-reactive astrocyte in exosomes-treated SOD1(G93A) mice was evident, although the difference with PBS-treated mice was not significant (Figure 6A). Interestingly, i.n. exosomes-treated mice showed a trend of astrocytosis reduction as compared with the PBS group at week 15. This reduction became significantly different at week 19 (*p* = 0.03, Figure 6B,C), demonstrating the effect of i.n. exosomes treatment on reducing astrocyte activation at the late stage of ALS disease (Figure 6C).

Concerning the number of microglial cells, no difference was observed in the lumbar spinal cord of exosomes- and PBS-treated mice, at any time points.

### 2.6. ASC-Exosomes Selectively Reach the Lesioned Area of the Brain in SOD1(G93A) Mice

To demonstrate the ability of ASC-exosomes to reach the CNS, labeled exosomes-USPIO were administered intranasally in SOD1(G93A) mice at 13 weeks of life, at the time when typical ALS brain lesions are detected by MRI [15,16].

**Figure 7:**
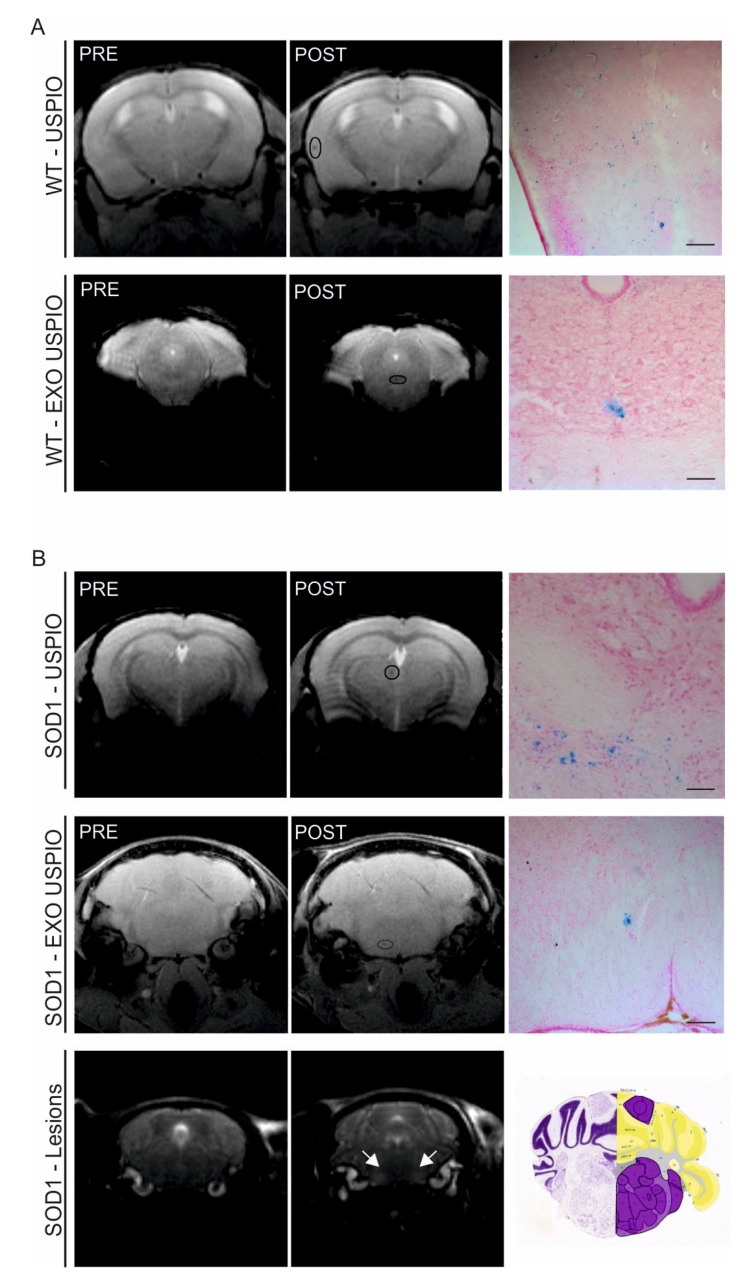
In vivo MR images and PB staining of the WT and SOD1(G93A) mice brain. (**A**) Representative in vivo MR images acquired prior (left) and 3 h post (center) i.n. administration of ultra-small superparamagnetic iron oxide nanoparticles (USPIO) nanoparticles (first line) or exosomes labeled with USPIO (EXO USPIO, second line). On the right, the PB histological examination of the brain shows blue spots in the same area revealed by MRI, confirming the presence of USPIO nanoparticles (magnification 20×, scale bar 50 µm). Both USPIO and exosomes-USPIO are randomly distributed in the brain; (**B**) In the first and second lines, representative MR images acquired pre (left) and 3 h post (center) i.n. administration of USPIO nanoparticles or exosomes labeled with USPIO (EXO USPIO). On the right, the PB histological examination of the brain shows blue spots in the same area revealed by MRI, confirming the presence of USPIO nanoparticles (magnification 20×, scale bar 50 µm). Note that, while USPIO nanoparticles are randomly distributed in the animal brain (USPIO were detected in different areas in different mice and the images reported in the first line is a representative one), exosomes-USPIO selectively reached the lesioned ALS area in all the mice analyzed (compared the second line with the third line). In the third line, representative T2w MR images performed to identify typical lesioned area of the SOD1(G93A) mice (arrows). The analysis revealed a signal intensity enhancement compared with surrounding tissue, indicating the cell degeneration during the progression of the disease. The region of the coronal mouse brain map corresponding to the MRI image is reported on the right.

The MR images revealed hypointense spots in the brain after 3 h of administration, attributable to the presence of USPIO nanoparticles or exosomes-USPIO. In the WT mice, T2*-weighted images of the brain pre and 3 h post i.n. administration of USPIO or exosomes-USPIO revealed a signal that was randomly distributed in the brain (Figure 7A). Blue spots after Prussian blue histological analysis also confirmed the presence of USPIO nanoparticles in the same cerebral areas revealed by MRI (Figure 7A). These data indicate that ASC-exosomes delivered intranasally home to the brains of the WT mice. However, since the WT mice were healthy animals, ASC-exosomes were not recruited in a specific cerebral area, as demonstrated by our analysis in which the signal was randomly distributed in the brain. Indeed, in all the analyzed WT brain sections, the exosomes-USPIO signal was detected in different brain areas. The image presented in Figure 7A is a representative image obtained from one mouse.

In the SOD1(G93A) mice, high-resolution T2 map MR images were performed to identify the lesioned areas, localized in brainstem regions corresponding to the trigeminal and facial motor nuclei (Figure 7B), according to the literature [15,16]. The T2*-weighted MR images obtained in the SOD1(G93A) mice pre and 3 h post i.n. administration of only USPIO showed that nanoparticles are randomly distributed in the brain, as in the WT mice (Figure 7B); in all the analyzed brains of the SOD1(G93A) mice that received only USPIO, the signal was detected in different brain areas. Interestingly, in the SOD1(G93A) mice that received exosomes-USPIO, the MR images acquired 3 h post-administration and the subsequent histological staining showed that exosomes reached the injured area of the brains of the mice, indicating the ability of ASC-exosomes to home to the lesioned sites in ALS disease (Figure 7B).

No hypointense signal was detected in the brains of the mice after 24 h of exosomes-USPIO or USPIO nanoparticles administration, indicating a possible metabolization of iron after ASC-exosomes internalization by the cells.

## 3. Discussion

The development of new therapies for ALS is crucial to improve the quality of life of patients and extend their survival. Many studies have been conducted in order to develop pharmacological/cell therapies [17,18,19], but the challenge is still open. It is known that ALS is a multifactorial disease, since different pathogenetic mechanisms are involved in motoneuron degeneration [3]. All pharmacological treatments tested for ALS have usually been focused on single or few altered cellular pathways, and all clinical trials have failed to show any significant results [5]. The stem cell therapy has the advantage that it can act via multiple mechanisms, simultaneously counteracting different pathogenetic pathways and providing a more effective treatment for ALS patients [3]. The therapeutic efficacy of stem cells of different origins has been evaluated in experimental models of ALS, with encouraging results [7,8,9,10].

It has emerged that the beneficial function of MSC could be mediated by the release of extracellular vesicles, as exosomes, rather than their engraftment and differentiation [20]. A therapy based on the use of exosomes would prevent all the risk associated with cells transplantation and would require administering only the fraction responsible for the beneficial effect of stem cells [21]. The exosomes transfer functional molecules (in particular proteins, mRNA, and miRNA) to other cells, modulating their activity, and maintaining the integrity of their cargos from proteases and nucleases degradation. Moreover, among the extracellular vesicles, exosomes are the only ones that can cross the blood-brain barrier thanks to their small dimension, a feature that renders these vesicles more interesting as a therapeutic approach in several neurodegenerative diseases [13]. With evidence that extracellular vesicles recapitulate the effect of stem cells, several works have tested their therapeutic efficacy in neurodegenerative disease models. The exosomes reduce the pathological accumulation of the β-amyloid peptide in an in vitro model of Alzheimer’s disease stimulating their proteolysis [22], protecting dopaminergic neurons from apoptosis after oxidative stress [23], and showing a neuroprotective effect in an in vitro model of ALS [24]. In this regard, the proteomic analysis of exosomes, correlating the protein content to the anti-apoptotic effect, was useful to understand the mechanisms by which ASC-exosomes exert their beneficial effect [25]. In the in vivo model of the disease, exosomes are able to promote neural plasticity and functional recovery after stroke in rats via the transfer of miRNA-133b [26], induce neurite outgrowth and improve neuroregeneration after traumatic brain injury in C57BL6 mice [27], and ameliorate chronic experimental autoimmune encephalomyelitis [28]. Moreover, exosomes isolated from a different source of MSC alleviate inflammation and prevent cognitive and memory impairments after status epilepticus [29], alleviate neuroinflammation and reduce β-amyloid accumulation in a mouse model of Alzheimer’s disease [30], and suppress inflammatory response enhancing the regeneration of mice spinal cord after injury [31].

In the present study, we investigated whether ASC-exosomes exert a protective effect in vivo in transgenic SOD1(G93A) mice of ALS. We decided to administer ASC-exosomes every four days, since this experimental paradigm presents an optimal compromise between frequency and route of administration, above all for chronic intravenous administration in which more frequent injections could be harmful to the mice and not allow the regeneration of epithelial vein tissue. We demonstrated that repeated i.v. or i.n. administrations of ASC-exosomes improved the motor performance from week 11 to week 15 of life as compared with the controls (PBS-treated mice). From week 17 of life, the beneficial effect observed in ASC-exosomes treated mice disappeared and no differences in motor performance were observed as compared with the PBS group. This data indicates that, at the late phase of the disease, when the interaction between MN and NMJ is highly compromised [32], the dose of ASC-exosomes used was inefficient, underling that this treatment could be dose-dependent, as already demonstrated in other experimental studies [33,34,35].

As reported in the literature, in SOD1(G93A) mice, there is a progressive lumbar MN degeneration during the progression of the pathology [36]. This result is also confirmed by our study, which showe 22.9% MN loss at week 15 and 56.2% MN loss at week 19. Interestingly, we demonstrated that ASC-exosomes treatment was able to protect lumbar MN degeneration during the disease progression, showing a progressively increased percentage of MN survival between 15 and 19 weeks of life, being significant at the last time point. In addition to survival, our data suggest that ASC-exosomes improves the function of MN, as indicated by a significantly higher number of preserved NMJ and skeletal muscle fibers at 15 weeks of life.

Neuroinflammation, determined by astrogliosis, microgliosis, and infiltration of immune cells, is a common characteristic of ALS and other neurodegenerative diseases. In particular, through the release of soluble factors, astrocytes exert a potent toxic property on MN, contributing to their neurodegeneration [37,38]. It has been reported that exosomes derived from stem cells suppress the activation of neurotoxic astrocytes after traumatic spinal cord injury, ischemia, and epilepsy [31,39,40]. In our study, we demonstrate that ASC-exosomes are able to decrease astrocytes activation but not the total number of activated microglial cells.

The route of delivery of ASC-exosomes could also have profound relevance. In this study, we tested the intranasal administration, which represented a noninvasive procedure that allowed repetitive dispensation and an efficient delivery of the vesicles to the brain. These characteristics make the i.n. administration an efficient approach to deliver exosomes to the regions involved in this neurological disease, since earlier studies have shown that the systemic administration of exosomes determined an accumulation in the spleen and the liver [41,42]. Moreover, these features would allow an easy and more effective transferability of ASC-exosomes treatment for patients with neurological diseases.

To understand the migration and homing abilities of exosomes in the brain after i.n. administration, a recent study reported that bone marrow MSC-exosomes selectively targeted lesioned areas in murine models of stroke, Parkinson’s disease, Alzheimer’s disease, and autism [43]. In this study, we investigated the distribution of ASC-exosomes by MRI after 3 and 24 h of i.n. administration. We demonstrated that ASC-exosomes reached the injured brain area of SOD1(G93A) mice, the brainstem regions corresponding to the trigeminal and the facial motor nuclei [15,16]. This indicates that injured signals could play a role in attracting ASC-exosomes in the specific injured brain for a limited period of time. Indeed, hypointense signal indicating the presence of labeled exosomes was not detected after 24 h. This could indicate that ASC-exosomes spread through the CNS and reach the lesioned spinal cord. Data concerning the homing of ASC-exosomes in the spinal cord could elucidate if their action is direct on MN and astrocyte in this site of CNS. Moreover, it remains to be clarified which cells uptake ASC-exosomes. The elucidation of these mechanisms would contribute to open the way to the application of exosomes therapy in ALS and other neurodegenerative conditions.

## 4. Materials and Methods

### 4.1. ASC Cell Cultures

Murine ASCs were obtained from inguinal adipose tissues of C57Bl6/J mice (Charles River Laboratories, Sant’Angelo Lodigiano, Italy). The isolation of stromal vascular fraction was carried out as previously described [44]. The extracellular matrix was incubated in Hank’s Balanced Salt Solution (Life Technologies Italia, Milan, Italy) with collagenase type I (Life Technologies Italia, Milan, Italy) and bovine serum albumin (BSA, AppliChem Nova Chimica Srl, Milan, Italy), centrifuged, and suspended in NH_4_Cl. The fraction was centrifuged again and filtered to remove cell debris. Then, cells were cultured using DMEM, 10% FBS, 100 U/mL penicillin, and 100 μg/mL streptomycin (all from GIBCO Life Technologies, Milan, Italy) and incubated at 37 °C/5% CO_2_. Murine ASCs were recognized by immunophenotype using monoclonal antibodies specific for CD106, CD9, CD44, CD80, and CD138, and by the absence of hematopoietic and endothelial markers (as CD45, CD11c, and CD31), as previously described [8].

### 4.2. ASC-Exosomes

Exosomes were isolated from the culture medium of 10^7^ASC. The cells were cultured to confluence and 48 h of FBS deprivation was made to avoid any contamination of vesicles from serum.

To isolate labeled exosomes-USPIO, 10^7^ASC were cultured to confluence, and then incubated with 200 µg Fe/mL of USPIO for 24 h, followed by FBS deprivation for 48 h, as previously described [14].

Then, Ccll culture supernatant was collected and exosomes (labeled or unlabeled) were obtained using Pure Exo Exosomes Isolation Kit (101Bio, Montain View, CA, USA), following the manufacturer’s protocol. The protein content of exosomes was determined by Bicinchoninic Protein Assay using the manufacturer’s protocol (Thermo Scientific™, Milan, Italy BCA™ Protein Assay). The size and concentration of ASC-exosomes were assessed by QNano instrument.

Concerning exosomes-USPIO, the quantification of iron nanoparticles was performed as previously described in [45].

The ASC-exosomes were used to evaluate their neuroprotection in vivo, the exosomes-USPIO were used in MRI to detect their presence in the CNS after in vivo administration.

### 4.3. Electron Microscopy and Western Blot

Electron microscopy of exosomes was performed as previously described [24]. Exosome pellets were fixed in 2% glutaraldehyde in Sorensen buffer (pH 7.4) for 2 h, and then postfixed in 1% osmium tetroxide (OsO_4_) in aqueous solution for 2 h. The sample was dehydrated in graded concentrations of acetone and embedded in Epon-Araldite mixture (Electron Microscopy Sciences, Fort Washington, PA, USA). The semithin sections (1 µm in thickness) were examined by light microscopy (Olympus BX51, Olympus Optical, Hamburg, Germany) and stained with toluidine blue. The ultrathin sections were cut at a 70 nm thickness, placed on Cu/Rh grids with Ultracut E (Reichert, Wien, Austria), and observed with transmission electron microscopy (TEM) using a Morgagni 268D electron microscope (Philips).

Western blot hybridization was performed as previously described [24]. Proteins were denatured, separated on 4%–12% polyacrylamide gels and transferred onto a nitrocellulose membrane. Antibodies against murine HSP70 (70 kDa, 1.100 HSP70 (K-20): sc-1060 Santa Cruz Biotechnology, DBA Italia Srl, Milan, Italy) and CD9 (25 kDa, 1:100 MM2/57, Millipore CBL-162) were used, followed by HRP-conjugated secondary antibodies (Dako Agilent, Milaln, Italy). ASC lysates were used as a positive control. Then, the blot was incubated with a chemiluminescent HRP substrate and detected with G:BOX F3 GeneSys (Syngene, Cambridge, UK).

### 4.4. Animals

Experiments were performed using transgenic mice overexpressing human SOD1 carrying a Gly93-Ala mutation (SOD1(G93A)) (strain designation B6SJL–TgN[SOD1–G93A]1Gur, stock number 002726) and wild-type (WT) mice (B6SJL) obtained from Jackson Laboratories (Bar Harbor, ME, USA). Animals were maintained under controlled environmental conditions (temperature, humidity, 12 h/12 h light/dark cycle, water, and food ad libitum) and veterinarian assistance. The research complies with the commonly accepted “3Rs”. The experiments were performed with the approval of the Animal Care and Use Committee of the University of Verona (CIRSAL), and the Italian Ministry of Health, in strict adherence to the European Communities Council (2010/63/EEC) directives (project identification code 710/2018-PR, approved 24/09/2018), minimizing the number of animals used and avoiding their suffering. Transgenic mice were identified by a polymerase chain reaction specific for human SOD1 gene (primers for SOD1 gene were forward (113) 5′-CATCAGCCCTAATCCATCTGA-3′ and reverse (114) 5′-CGCGACTAACAATCAAAGTGA-3′; while for the housekeeping gene interleukin-2 receptor (IL-2R) the primers were forward (42) 5′-CTAGGCCACAGAATTGAAAGATCT-3′; reverse (43) 5′-GTAGGTGGAAATTCTAGCATCATCC-3′).

### 4.5. Motor Tests

The progression of the disease was monitored starting at 50 days by careful motor performance examination. In order to test the efficacy of the treatment, SOD1(G93A) mice were weekly evaluated blinded for body weight, neurological score test, paw grip endurance (PaGE) test, and rotarod test. The neurological score test was evaluated as follows: 4, normal (no sign of motor dysfunction); 3, hind limb tremors were present when the mice were suspended by tail; 2, gait abnormalities; 1, dragging at least one hind limb; and 0, inability to right itself in 30 sec when the animal was placed in the supine position. The PaGE test was used to assess the grip strength of the animals. The test was performed by placing the animal on a metal grid and quickly turning it over. The score was measured by the length of time that the mouse was able to grip onto the grid. Each mouse was given up to two attempts to hold onto the inverted grid with an arbitrary cut-off time of 120 s. The rotarod test was used to assess the motor coordination of the animals. The mice were placed in a rotor tube (Acceler Rota-Rod 7650, Ugo Basile, Varese, Italy) at a constant speed of 16 rpm. The cut-off time was settled at 180 sec and three attempts were given to mice that failed the test, with a resting phase of 5 min. The longest latency time was registered.

The animals failed the PaGE test or the rotarod test when they were not able to reach the cut-off time. The onset was established when the mouse failed the PaGE or rotarod test. When the neurological score was equal to zero, the animals were sacrificed, and the survival time was recorded.

### 4.6. Animal Treatments

To test the therapeutic efficacy of ASC-exosomes, a total of 56 SOD1(G93A) male mice were used in the experimental paradigm. The ASC-exosomes were administered in SOD1(G93A) mice intravenously (10 treated mice and 10 control mice) or intranasally (10 treated mice and 10 control mice). The ASC-exosomes administrations were performed from the onset of the clinical sign until the end stage, every 4 days. For each injection, the treated mice were injected with 1 µg of ASC-exosomes, while the control mice received sterile PBS. The total amount of injected solution was 100 µL for an intravenous injection and 10 µL for intranasal administration.

A third group of animals was used. These SOD1(G93A) mice (8 treated mice and 8 control mice) received ASC-exosomes intranasally every 4 days but, in this case, the administrations were performed from the onset of the clinical sign until 15 weeks of life. This time point represents an intermediate point between the onset and the end stage of the disease, when the motor performance was statistically significant different between the control versus the treated groups.

### 4.7. Lumbar Spinal Cord Motoneurons Stereological Count

At the end stage (19 weeks) or at 15 weeks of life, the SOD1(G93A) mice (*n* = 5 per group) were deeply anesthetized and transcardially perfused with PBS 0.1 M followed by paraformaldehyde 4%. The spinal cord was dissected out and 2 h of post-fixation was performed. The lumbar tract was soaked in 30% sucrose, included in OCT and serially cut at 15 µm with cryostat apparatus. The sections were mounted on Surgipath^®^ Apex™ Superior Adhesive Slides (3800080E, Leica Biosystems Italia, Milan, Italy).

For Nissl staining, the slides were air-dried, and then hydrated with H_2_O for 30 sec. The sections were stained with 0.2% cresyl violet solution for 8 min and gradually placed into increasing concentrations of ethanol, cleared with xylene, mounted with Entelan and covered with a cover glass. The MN of the ventral horn in the lumbar spinal cord tract (lateral and medial MN of L1-L5 segments) were counted blinded every 100 µm (every 6/7 slide) by the operator using a computer-assisted microscope (Olympus BX6 with Retiga 2000R camera, Center Valley, PA) with the Stereoinvestigator software (MicroBrightField, Williston, VT, USA) at 40× magnification. Cells with nucleoli on the plane of focus, size and shape typical of MN were counted. The values from the sections were computed for the summation, the mean number was then computed from the average number derived from each animal.

### 4.8. Immunohistochemistry of Lumbar Spinal Cord

To investigate the activation of astrocytes and microglia cells in the lumbar tract of the spinal cord, immunohistochemistry for light microscopy was performed. The sections were incubated for 10 min in 3% H_2_O_2_ to quench endogenous peroxidase and preincubated for 1 h in 5% of NGS in PBS and 1% BSA. The slides were incubated overnight in anti-mouse GFAP or Iba1 antibodies to recognize astrocytes and microglia, respectively, (GFAP 1:500, Z0334 Dako; Iba1 1:500 019–19741 Wako) in 1% NGS in PBS. The sections were washed and incubated for 1 h in biotinylated goat anti-rabbit IgG (1:100, Vector Laboratories). The avidin-biotin peroxidase kit (ABC kit; Vector) and Novared kit (Vector) as signal revelation system was used. After mounting on slides, the sections were dehydrated through increasing grades of ethanol, cleared in xylene, and coverslipped with Entellan (Merck, Darmstadt, Germany). For the analysis, cells were counted every 100 µm for a total of 30 sections for each animal. The astrocytes and microglial cells (GFAP or CD11b labeled cells) of the lumbar tract (L1-L5) were visualized and counted using a computer-assisted microscope (Olympus BX6 with Retiga 2000R camera) with the Stereoinvestigator software (MicroBrightField, Williston, VT, USA).

### 4.9. Immunohistochemistry of Neuromuscular Junction and Hematoxylin-eosin Staining

WT (*n* = 5) and SOD1(G93A) mice PBS- or exosomes-treated (*n* = 5 per group) were deeply anesthetized and transcardially perfused with PBS 0.1 M followed by paraformaldehyde 4%. The hindlimb gastrocnemius muscle was dissected out, post-fixed for 2 h, soaked in 30% sucrose, included in OCT, and longitudinally cut at 20 µm with cryostat apparatus. The sections were mounted on Surgipath^®^ Apex™ Superior Adhesive Slides (3800080E, Leica Biosystems Italia, Milan, Italy). The sections were washed with PBS Triton for 20 min and stained for postsynaptic acetylcholine receptors using CF543 conjugates α-bungarotoxin (αBTx, 1:500, Biotium, DBA Italia Srl, Milan, Italy) for 25 min at room temperature. The sections were washed and incubated for 1 h in 10% NDS and 0.3% Triton X-100 in PBS. Presynaptic motor terminals were labeled using anti-neurofilament H (NF-H; 1:100, Chemicon, Merck, Milan, Italia) overnight at 4 °C. After washing, the sections were incubated with species-specific Alexa Fluor 488 secondary antibody in 2% NDS in PBS (1:1000; Invitrogen, Thermo Fisher Scientific, Milan, Italy) for 1 h at room temperature. After washing, the sections were incubated with DAPI (1:1000; Santa Cruz Biotechnology, DBA Italia Srl, Milan, Italy) for 3 min at room temperature. Sections were washed and mounted with Dako Fluorescence Mounted Medium (Agilent, CA; USA) and dried prior to analysis.

For hematoxylin-eosin (HE) staining, the hindlimb gastrocnemius muscle was transversely cut at 20 µm with cryostat apparatus. The sections were stained with hematoxylin for 40 s, washed with tap water, immersed in eosin for 30 s, and washed with tap water. The sections were dehydrated in ascending alcohol solutions, cleared with xylene, and coverslipped with Entellan (Merck, Darmstadt, Germany). The sections were visualized by optical microscopy (Olympus BX63; Olympus Life Science Solutions, Center Valley, PA). A total of 100 fibers were examined for each animal. The image J software was used to measure the area of gastrocnemius muscle fiber in each group.

### 4.10. Statistical Analysis

Concerning the motor performance, two-way univariate analysis of variance (ANOVA) and Bonferroni post-hoc tests were performed to evaluate differences between the two animal groups. Data are expressed as mean ± standard error of the mean (SEM). Survival data of the animals were analyzed by Gehan–Breslow–Wilcoxon test. The stereological MN count, immunohistochemistry analysis of glial cells, and data of NMJ immunohistochemistry were analyzed using a two-tailed Student’s test to evaluate differences between groups. Data are reported as mean ± SEM. For all statistical analysis and graphs, GraphPad Prism 5 Software was used, and significance was accepted at *p* < 0.05.

### 4.11. Exosomes-USPIO or USPIO Administration

To investigate the ASC-exosomes migration in the CNS, the WT mice (*n* = 8) and SOD1(G93A) mice (*n* = 8) were divided into the following two groups: one group that received exosomes-USPIO and one group that received only USPIO nanoparticles. The administration was performed at 13 weeks of life in SOD1(G93A) mice, and at the same time of life in the WT mice. This time represents the time point when the typical ALS brain lesions are reported by MRI [15,16].

The exosomes-USPIO were administered intranasally at a concentration of 30 µg of exosomal proteins (containing 0.2 µg of Fe), resuspended in 20 µL of PBS. In the animals that received only USPIO nanoparticles, an amount of USPIO containing the same quantity of Fe present in exosomes-USPIO was administered.

### 4.12. In Vivo MRI

MR images were acquired using a Bruker Tomograph (Bruker, Karlsruhe, Germany) equipped with a 4.7 T, 33 cm bore horizontal magnet (Oxford Ltd., Oxford, UK). The SOD1(G93A) and WT mice were anesthetized using 1% isofluorane inhalation in a mixture of oxygen and nitrogen and were placed in a prone position over a heated bed. Brain images were acquired with a cross-coil configuration, i.e., a volume birdcage coil was used as transmitter, and a surface helmet coil specific for the mouse brain was used as a receiver. The MRI acquisitions were performed before i.n. administration in all animals. At 13 weeks of life, the SOD1(G93A) mice received i.n. USPIO or exosomes-USPIO (6 animals for each group). The WT mice received i.n. USPIO and exosomes-USPIO (6 animals for each group) at the same weeks of life as the SOD1(G93A) mice. The MRI acquisitions were performed after 3 and 24 h of i.n. administration.

In order to detect USPIO and exosomes-USPIO, T2* map images were acquired using a Multi Gradient Echo (MGE) sequence with repetition time (TR) = 2000 ms, echo time (TE) = 3.6 ms, field of view (FOV) = 2 × 1.5 cm^2^, matrix size (MTX) = 160/120, slice thickness = 0.5 mm; n° of slice = 20°; number of averages = 9; total acquisition time = 27 min.

Moreover, only in the SOD1(G93A) mice, to identify the areas of lesion, high-resolution T2 weighted structural images were acquired using a Rapid Acquisition with Refocused Echoes (RARE) sequence with the following parameters: TR = 5000 ms; TE = 76 ms; FOV = 2.5 × 2.0 cm^2^; MTX = 160 × 128; slice thickness = 0.5 mm; n° of slice = 20°; RARE factor = 16; number of averages = 24.

### 4.13. Histological Analysis after Exosomes-USPIO Administration

After in vivo administration of exosomes-USPIO and MRI acquisitions, the presence of USPIO nanoparticles in the brain of the animals, indicated the presence of exosomes, which was confirmed by histological analysis. Mice were sacrificed and the brain was dissected out, fixed in paraformaldehyde 4%, and soaked overnight for cryoprotection in 20% sucrose at 4 °C. All the samples were cut on a cryostat, and transverse sections (25 µm thick) were collected from all the brains. In order to evaluate the presence of USPIO nanoparticles, Prussian blue staining was performed. The sections were incubated with Prussian blue solution (5% hydrochloric acid and 5% potassium ferrocyanide) for 40 min and counterstained with nuclear fast red, for 10 min. The sections were dehydrated through increasing grades of ethanol, cleared in xylene, coverslipped with Entellan (Merck, Darmstadt, Germany), and examined under a light microscope (Olympus BXS1, Center Valley, PA) equipped with a CCD camera. The sections were examined with the brain mouse atlas in order to confirm that the area detected by histological analysis overlapped with the one detected by MRI.

## 5. Conclusions

In the present study, we tested the effect of two different routes of ASC-exosomes administration (i.v. or i.n.) on SOD1(G93A) murine model of ALS, and demonstrated that repeated administration improved the motor performance; protected lumbar motoneurons, the neuromuscular junction, and muscle; and decreased the glial cells activation in the exosomes-treated animals. We maintain that the i.n. delivery could easily be transferable to ALS patients and represents an innovative route for the promising application of exosomes in this neurodegenerative disease. Furthermore, i.n administration is a noninvasive way to deliver exosomes directly to the CNS, a fundamental feature to cure neurological disorders. The data reported in this study contributes by providing additional knowledge for the promising use of ASC-exosomes as a therapy in ALS and other neurodegenerative diseases.

## Abbreviations

ALS	Amyotrophic lateral sclerosis
ASC	Adipose-derived stem cells
ASC-exosomes	Exosomes isolated from ASC
αBTx	α-bungarotoxin
Exosomes-USPIO	Exosomes labeled with ultra-small superparamagnetic iron oxide nanoparticles
i.n.	Intranasal
i.v.	Intravenous
MN	Motoneurons
MNJ	Neuromuscular junction
MRI	Magnetic resonance imaging
MSC	Mesenchymal stem cells
PaGE	Paw grip endurance test
SOD1	Cu^2+/^Zn^2+^ superoxide dismutase
SOD1(G93A)	Human SOD1 gene with a G93A mutation
USPIO	Ultra-small superparamagnetic iron oxide nanoparticles
